# Effects of Fortified Wheat Bran Arabinoxylan on the Quality of Wheat Malt Beer

**DOI:** 10.3390/foods14061036

**Published:** 2025-03-18

**Authors:** Kai Jiang, Yuhong Jin

**Affiliations:** College of Food Science and Engineering, Shandong Agricultural University, Tai’an 271002, China; jk15969823103@163.com

**Keywords:** wheat beer, wheat bran, arabinoxylan, molecular weights, structure–property modeling

## Abstract

Arabinoxylan, a key non-starch polysaccharide in wheat bran, significantly influences the quality and health benefits of wheat beer. This study aimed to investigate how wheat bran addition (0–20%) affects water-extracted arabinoxylan (WEAX) content and beer quality in 100% wheat malt beer. The study integrated physicochemical analyses (polysaccharide composition, WEAX molecular weight), process parameters (wort filtration time, foam stability), and sensory evaluation to establish structure–function relationships. Results showed that the WEAX content in beer increased from 1.36 mg/mL in pure malt beer (0% bran) to 2.25 mg/mL with 20% bran addition. Bran addition shortened wort filtration time by 20–45%. The molecular weight of WEAX was mainly 2936–7062 Da, enhancing foam expansion (36.18%) and stability (15.54%) due to elevated polymerization and arabinose-to-xylose (A/X) ratios. WEAX fractions (7062–10,134 Da and 859–2936 Da) correlated positively with beer turbidity and viscosity. Sensory analysis identified 15% bran as optimal for balanced quality. These findings demonstrate that bran addition enhances WEAX content, polymerization, and A/X ratios, improving foam performance, reducing filtration time, and optimizing beer quality without altering arabinogalactan, glucan, or mannose polymer content.

## 1. Introduction

Beer has long been the most popular alcoholic beverage globally [1]. Recently, wheat beer has experienced a surge in popularity within the Chinese beer market due to its frothy foam, hazy appearance, aromatic phenolic notes, and robust flavor, establishing itself as a beloved beer choice. Wheat beer, made by incorporating wheat alongside barley malt or using wheat as the primary ingredient, has a rich history. It encompasses various well-known styles like Weissbier, Krystal Weisse, Hefe Weisse, and Witbier [2,3].

Wheat malt is the main ingredient in wheat beer and contains non-starch polysaccharides, primarily arabinoxylan (AX). The AX backbone is formed by β-1,4 glycosidic linkages of D-xylose residues, with α-L-arabinofuranose substituents at the O-3 and/or O-2 positions of the xylose residues. The arabinose residues connected to the O-3 position of the main chain xylose residues are ester-linked to ferulic acid [4]. The content and properties of AX in wheat are determined by the genotype and environmental conditions. Generally, the AX content in wheat ranges from 1.52% to 6.36% [5], with an arabinose-to-xylose (A/X) ratio of approximately 0.6. However, the AX content in wheat bran is much higher than in wheat grains, reaching over 15% of the whole wheat kernel [6,7]. The AX content and structure vary in different parts of wheat bran. In the aleurone layer, AX accounts for 17.6% to 23.9%, with an A/X ratio of 0.38 to 0.47. In the intermediate layer, AX accounts for 37.4% to 40.0%, with an A/X ratio of 0.34 to 0.42. In the outer pericarp, AX accounts for 42.6% to 46.9%, with an A/X ratio of 1.13 to 1.19 [8]. Wheat malt contains a rich enzymatic system, including proteases, amylases, and hemicellulase enzymes. The hemicellulase enzymes include endo-xylanases, arabinofuranosidases, ferulic acid esterases, and glucanases. During the beer mashing process, hemicellulase enzymes work together to degrade AX in wheat, converting insoluble AX into soluble forms and breaking it down from large molecules into smaller ones. This process ultimately influences the content and molecular weight composition of AX in the wort and beer. Research indicates that WEAX in beer significantly impacts beer quality. When the WEAX content increases to 0.80 g/L, with an average degree of polymerization (avDP) reaching 50, it enhances the beer body and positively affects the turbidity and foam stability of barley beer [9]. When exogenous hemicellulase and β-glucanase are added during the mashing process, the content of high molecular weight arabinoxylan decreases by 23% to 58%, and beer viscosity decreases by 0.04 to 0.10 mPa·s [9,10]. Furthermore, AX exhibits various biological functions, including antioxidant activity, inhibition of tumor cell growth, promotion of gut microbiota abundance and diversity, and a regulatory effect on hypoglycemia [11,12,13,14]. Studying the relationship between the content of AX in wheat beer and its functional characteristics is significant for improving the quality of wheat beer and enhancing its health benefits. Additionally, wheat presents filtration challenges in the beer production process due to the absence of a filter bed. This study innovatively introduces wheat bran as a multifunctional additive to simultaneously enhance AX-mediated beer quality (e.g., viscosity, foam stability) and mitigate AX-induced filtration challenges, resolving the inherent conflict between sensory richness and brewing efficiency through targeted AX modulation.

While wheat beer’s sensory traits (e.g., haze, foam stability) are linked to water-extractable arabinoxylan (WEAX) properties, the mechanistic impact of bran addition on WEAX molecular architecture remains unclear. This study elucidates bran-induced WEAX evolution (content, molecular weight, A/X ratio) during brewing and establishes quantitative structure–property models linking WEAX to viscosity, turbidity, foam dynamics, and rheological behavior. By resolving molecular restructuring mechanisms, we address the trade-off between filtration efficiency and sensory quality in high-viscosity wheat beer production.

## 2. Materials and Methods

### 2.1. Materials

The wheat bran was purchased from the Tai’an Agricultural Market, and the wheat malt was obtained from Yuehai Yongshuntai Malt Co., Ltd. (Guangzhou, China) The basic parameters of both wheat bran and wheat malt are listed in Table 1.

### 2.2. Beer Sample Preparation

The ratio of raw materials to water was 1:3.5, divided into five groups: A, B, C, D, and E. The quantities of wheat bran added were 0%, 5%, 10%, 15%, and 20% of the total amount of raw materials, respectively. When wheat bran was added, an equivalent amount of wheat malt was subtracted to maintain a consistent total raw material weight. The mash was initially heated to 40 °C and held for 60 min. Subsequently, the temperature was raised to 50 °C and maintained for 50 min. Following this, the temperature was increased to 63 °C and held for 30 min. Finally, the temperature was raised to 70 °C until the iodine reaction disappeared and then maintained at 78 °C for 10 min. The heating rate is 1 °C/min. After filtration and boiling for 50 min, the hopping amount was 1 g/L (Sazz, Chmel Spol), the wort concentration was adjusted to 12 °P, and ale yeast (Nottingham, Lallemand) was added at a ratio of 1 g/L. Fermentation was carried out at 20 °C and stored at 4 °C once the reducing sugar content stabilized.

### 2.3. Determination of Basic Physicochemical Indexes of Wort and Wheat Beer

Viscosity was assessed utilizing a Haake falling ball viscometer. Chroma, pH, total acidity, FAN (free amino nitrogen), protein, saccharification time, filtration time, original wort concentration, apparent degree of fermentation, turbidity, and alcohol content were determined based on the Analytica—European Brewing Convention (EBC) standards [15].

### 2.4. Determination of Beer Foam Properties

The foam stability, foam volume expansion rate, and foaming capacity of the beer were measured based on the method of Hu [16]. In short, the beer is sonicated at 20 °C for 20 min to remove gas, and 20 mL of the deaerated beer sample is taken. CO_2_ is continuously injected into the beer at a constant pressure of 0.1 MPa for 30 s. Subsequently, the post-expansion total beer volume, foam volume, and foam collapse time were recorded.

### 2.5. Determination of Free Monosaccharides and Polysaccharide Content

The crude polysaccharide and monosaccharide content were assessed using gas chromatography, following the methods and conditions outlined by Li et al. [17]. Samples (2 mL) were hydrolyzed with trifluoroacetic acid (0.7 mL, 6 M) at 100 °C for 3 h, cooled, and diluted to 5 mL. A 1 mL aliquot was reduced with NaBH_4_ (0.3 mL, 1 M) and NH_4_OH (0.3 mL, 12 M) at 40 °C (1 h), quenched with glacial acetic acid (0.4 mL), then acetylated using 1-methylimidazole (0.5 mL) and acetic anhydride (4.5 mL, 10 min). GC Conditions (gas chromatograph GC-2030, Shimadzu, Kyoto, Japan): DM-2330 capillary column (30 m × 0.32 mm × 0.2 μm, Agilent Technologies, Santa Clara, CA, USA); 2.0 μL injection; injector 250 °C; EID detector 260 °C; oven 240 °C; nitrogen carrier gas (split ratio 14:1, 50.0 kPa); total flow 40 mL/min; 20 min runtime. Arabinose (Ara), xylose (Xyl), galactose (Gal), glucose (Glu), and mannose (Man) were used as standard sugars. The monosaccharide standard curves are presented in Table 2.

### 2.6. Determination of Molecular Weight of Polysaccharides

The molecular weight of polysaccharides was assessed using high-performance liquid chromatography (HPLC), following the methods and conditions outlined by Peng et al. [18].

The wort and beer samples were filtered through 0.45 μm membranes, mixed with four volumes of anhydrous ethanol (1:4, *v*/*v*), and precipitated at 4 °C for 24 h. After centrifugation (5000 r/min, 15 min), the pellet was redissolved and sequentially filtered through 0.45 μm and 0.22 μm membranes.

HPLC was used for determination, with a differential detector as the detector (RID-10, Shimadzu, Kyoto, Japan). The protective column is TSK (35 mm × 4.6 mm i.d.), and the chromatographic columns are TSKgel G3000 PWxl (300 mm × 7.8 mm i.d., Tosoh Co., Ltd., Tokyo, Japan) and TSKgel G4000 PWxl (300 mm × 7.8 mm i.d., Tosoh Co., Ltd., Tokyo, Japan). The sample size is 10 μL, the flow rate is 0.3 mL/min, the temperature is 40 °C, the analysis time is 70 min, and the mobile phase is 50 mmol/L sodium nitrate solution. Pullulan (1 g/L) was employed as the polysaccharide standard with molecular weights of 342, 1320, 6200, 10,000, 48,800, 113,000, 348,000, and 805,000 Da.

### 2.7. Sensory Analysis

The sensory analysis protocol was approved by the Ethics Committee of Shandong Agricultural University Animal Care and Use Committee (Approval No. SDAU 18-096) in compliance with the Declaration of Helsinki.

Eleven trained panelists assessed beers stored at 8 °C using the beer evaluation method criteria outlined in Mikyška [19]. Samples were equilibrated to 10–12 °C, served in ISO-standard tasting glasses (250 mL) under controlled lighting (D65 illuminant) and ambient noise (<40 dB). The sequential evaluation included visual analysis: clarity (haze scale: 0–3) and foam stability (collapse time measured over 5 min); aroma profiling: intensity (0: undetectable–5: intense) and quality (off-flavors, malt/hop balance); and taste/mouthfeel: quantified via 0–5 scales for sweetness (vs. sucrose reference), bitterness (vs. iso-α-acid standards), carbonation (tingling intensity), and body (perceived viscosity). Panelists are following a sip-and-spit protocol to minimize sensory fatigue.

### 2.8. Statistical Analysis

Each experiment and analytical measurement was repeated three times for accuracy. The data analysis and visualization were carried out using the software tools Microsoft Excel 2023, IBM SPSS Statistics 26, and Origin 2022. The significance of differences was assessed at a 95% confidence level with Tukey’s HSD test in IBM SPSS Statistics 25. Pearson correlation was used, with significance levels set at less than 0.05 for significant results and less than 0.01 for extremely significant results.

## 3. Results and Discussion

### 3.1. Influence of Bran Addition on Wort Fractions

#### 3.1.1. Influence of Bran Addition on the Sugar Fraction of Wort

**Figure 1:**
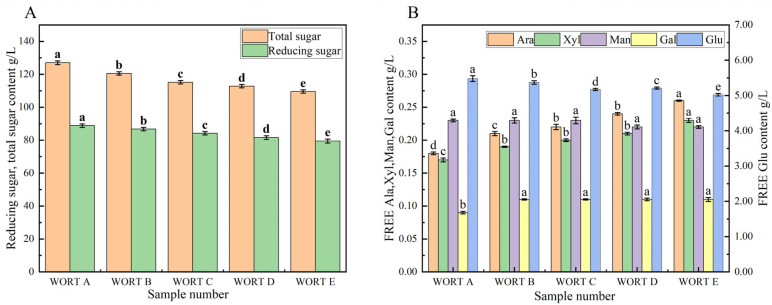
Effect of bran addition on the composition of reducing sugars, total sugars, and free monosaccharides in wort. (**A**) Reducing sugar and total sugar content; (**B**) free monosaccharides component (note: Ara: arabinose, Xyl: xylose, Man: mannose, Gal: galactose, Glu: glucose. Samples: WORT A (0% bran), B (5%), C (10%), D (15%), E (20%). Difference letters indicate significant differences between wort samples with different bran additions (*p* < 0.05).

The content of reducing sugars and total sugars in wort is presented in Figure 1A, while the content of free monosaccharides is shown in Figure 1B. In Figure 1A, it is demonstrated that with an increase in bran addition from 0% to 20%, the content of reducing sugars decreased from 88.85 g/L to 79.45 g/L. Similarly, the total sugar content decreased from 127.05 g/L to 109.55 g/L. In Figure 1B, Glu had the highest content among the free monosaccharides in wort, spanning 5.02 g/L to 5.48 g/L. The content of Ara, Man, Xyl, and Gal, respectively, exhibited the following ranges: 0.18–0.26 g/L, 0.22–0.23 g/L, 0.16–0.22 mg/mL, and 0.09–0.11 g/L. The content of Ara and Xyl in the wort significantly increased upon the addition of wheat bran with improvement rates of 44.44% and 35.29%, respectively. This increase may be traced back to the higher AX content in wheat bran and the consequent formation of more free Ara and Xyl in the presence of xylanase [20]. There was no significant change in the content of Gal and Man, while the Glu content decreased by 6.52%. As the bran addition increased from 0% to 20%, the content of Ara and Xyl continued to rise, indicating that the inclusion of wheat bran significantly increased the content of non-fermentable sugars in the wort.

The polysaccharide components and their contents in wort are shown in Table 3. The polysaccharide with the greatest amount in wort is water-extractable arabinoxylan (WEAX), followed by arabinogalactan (AG), β-glucan (GP), and mannose polymers (MP). The addition of wheat bran increased the WEAX content in wort from 1.41 to 2.42 mg/mL. There was negligible alteration in the content of AG and GP, while the content of MP increased from 0.23 to 0.29 g/L. As the proportion of bran increased, the solubilization rate of AX in wheat bran and the improvement rate of WEAX in wort showed a decreasing trend and then stabilized. The solubilization rate decreased from 31.78% to 21.17%. This might be attributed to the rapid increase in WEAX substrate in the system due to the addition of bran, which altered the optimal ratio of hemicellulase enzymes to substrates, thereby reducing the efficiency of hemicellulase enzyme degradation [21]. The addition of bran significantly increased the avDP of AX in the wort, with an increase of 19.17%. As the bran addition increased from 0% to 20%, The ratio of A/X rose from 0.52 to 0.67, indicating that the branching structure of AX in wort became more complex after the addition of bran [22]. The addition of wheat bran significantly increased the content of WEAX in the wort, reaching 1.02 g/L, which represents an improvement rate of 71.63%. It also elevated avDP and the A/X of WEAX in the wort. Additionally, the concentration of MP in the wort showed a marginal uptick of around 0.06 g/L.

#### 3.1.2. Changes in the Molecular Composition of Polysaccharides in Wort

**Figure 2:**
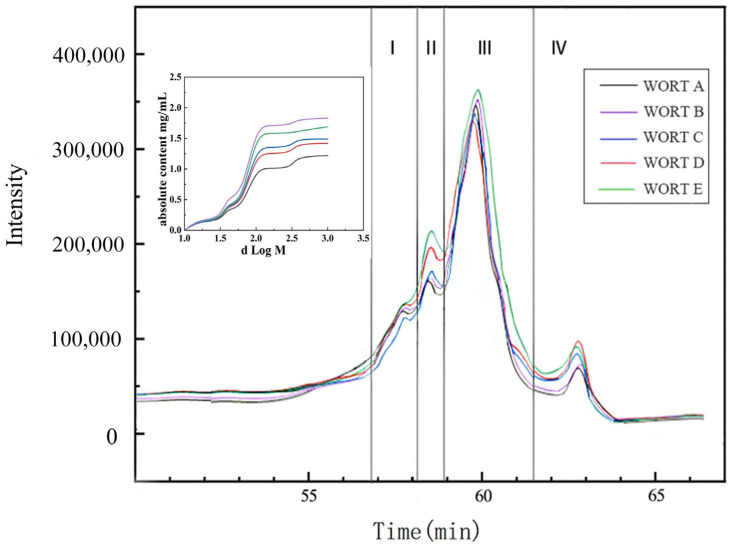
Molecular weight distribution and polysaccharide content in wort with different bran additions. I polysaccharide molecular weight 8411~10,959 Da; II polysaccharide molecular weight 7062~8411 Da; III polysaccharide molecular weight 3119~7062 Da; IV polysaccharide molecular weight < 920 Da. WORT A: adding 0% wheat bran; WORT B: adding 5% wheat bran; WORT C: adding 10% wheat bran; WORT D: adding 15% wheat bran; WORT E: adding 20% wheat bran.

The molecular weight and content of polysaccharides in wort with varying bran additions were analyzed using High-Performance Size Exclusion Chromatography with Refractive Index Detection (HPSEC-RID). Based on the peak shapes in Figure 2, the polysaccharides in wort were categorized into four components: I, II, III, and IV, with molecular weights ranging from 8411 to 10,959 Da, 7062 to 8411 Da, 3119 to 7062 Da, and <920 Da, respectively. Component III predominated in the wort, constituting 61.13% to 65.16% of the polysaccharide content, with absolute content ranging from 0.86 to 1.53 mg/mL. Following this, components I, II, and IV were the next most abundant, making up 16.12% to 16.87%, 12.32% to 13.31%, and 4.66% to 10.43% of the polysaccharide content, respectively. The absolute content of these components, respectively, varied between 0.23 and 0.40 mg/mL, 0.17–0.37 mg/mL, and 0.15–0.21 mg/mL.

As the addition of wheat bran increases, the peak heights of the four components in Figure 2 also continue to rise. It can be observed from Table 3 that the increased polysaccharides in wort are primarily due to WEAX. Therefore, the increase in peak heights of the four components in Figure 2 was mainly driven by the increase in WEAX. By combining the specific content of WEAX in the five types of wort from Table 3 with the relative content of each component peak in Figure 2, the molecular composition and absolute increase in WEAX in wort due to the addition of wheat bran can be calculated. The addition of wheat bran could enhance the content of WEAX in the four components (3119–7062 Da, 8411–10,959 Da, 7062–8411 Da, and <920 Da) from 0.23 to 0.67 mg/mL, 0.05 to 0.17 mg/mL, 0.05 to 0.14 mg/mL, and 0.02 to 0.07 mg/mL, respectively. Among these, the component with a molecular weight of 3119–7062 Da showed the most significant increase in content, with an improvement rate of 82.35%.

#### 3.1.3. Effect of Bran Addition on Physicochemical Indexes of Wort

The physicochemical parameters of the wort are listed in Table 4. It can be observed that with the increase in wheat bran addition from 0% to 20%, the free amino nitrogen (FAN) of the wort decreased. This change was primarily attributed to the lower protein content in wheat bran compared to malt. Additionally, with the addition of wheat bran, the viscosity of the wort rose from 1.96 to 2.37 mPa·s. Combining this information with Table 3, it is evident that the WEAX content in the wort increased by 66.67%, the avDP increased by 26.93%, and the A/X ratio increased by 26.92%. These findings further confirm that the higher content of WEAX, with a larger polymerization degree and more complex branching structure, is a significant factor influencing wort viscosity [23,24]. With the increase in wheat bran addition, the saccharification time of the wort increased from 8 min to 10 min. This can be attributed to two reasons. First, wheat malt exhibits a higher amylase content compared to wheat bran [25]. Secondly, the WEAX in wheat bran can competitively inhibit α-amylase activity, leading to prolonged saccharification time [26].

Based on the experimental results, it was observed that the filtration time was reduced from 40 min to 22 min with the addition of wheat bran, resulting in an improvement rate of 45.00%. This improvement was primarily attributed to the replacement of naked wheat. The fine sediment in naked wheat clogs the filter, making mash filtration difficult [27]. In contrast, wheat bran contains a significant amount of cellulose, which serves as a filtration bed and thereby enhances the filtration rate of the mash.

Due to a higher pigment content in wheat bran compared to wheat malt [28], as well as its richness in amino acids and other amino compounds, these substances serve as reactants in the Maillard reaction. When wheat bran undergoes high-temperature roasting and saccharification, the amino acids within it react with reducing sugars through the Maillard reaction. The resembling melanoidins were soluble in water and contributed to the chroma of the wort and the final beer [29,30,31]. Under the influence of these factors, the chroma of the wort increased from 6.3 EBC to 11.5 EBC.

### 3.2. The Effect of Wheat Bran Addition on Beer

#### 3.2.1. Influence of Bran Addition on the Sugar Fraction of Beer

**Figure 3:**
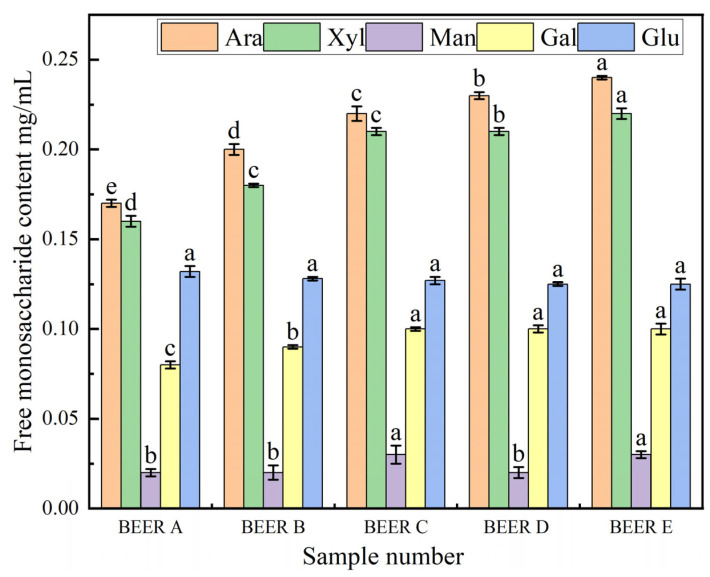
Types and contents of free monosaccharides in beer with different bran additions. Samples: BEER A (0% bran), B (5%), C (10%), D (15%), E (20%). Ara: arabinose, Xyl: xylose, Man: mannose, Gal: galactose, Glu: glucose. Difference letters indicate significant differences between beer samples with different bran additions (*p* < 0.05).

The content of free monosaccharides in beer is shown in Figure 3. Ara was the predominant free monosaccharide in beer, with concentrations ranging from 0.17 to 0.24 mg/mL, followed by Xyl (0.16–0.22 mg/mL), Glu (0.12–0.13 mg/mL), Gal (0.08–0.10 mg/mL), and Man (0.02–0.03 mg/mL). Adding wheat bran led to notable increases in Ara and Xyl levels in beer, with improvement rates of 41.18% and 37.50%, respectively. Combining this information with Figure 1, it can be observed that the levels of free Ara, Xyl, and Gal in beer were not significantly different from those in the wort. This was primarily because these sugars are non-fermentable and cannot be utilized by yeast during fermentation [32]. However, the utilization of yeast significantly reduced the levels of free Glu and Man [33], with reduction rates of 97.23% and 89.65%, respectively. When wheat bran was increased from 0% to 20%, the levels of Ara and Xyl in beer continued to rise, indicating that the inclusion of wheat bran notably elevated the concentration of non-fermentable sugars in beer.

The polysaccharide components and content in beer are presented in Table 5. It shows that the highest content of AX in beer was 1.36–2.25 mg/mL, followed by GP, AG, and MP, where the AG, MP, and GP content showed no significant changes among the five types of beer. With an increase in the addition of wheat bran, the avDP of AX in beer increased from 17.12 to 20.26, and the A/X increased from 0.53 to 0.66. During fermentation (comparing Table 3 and Table 5), the content of WEAX in beer remains relatively stable, with a decrease of 2% to 3%. The A/X ratio remained stable at around 0.5 to 0.6, and avDP ranged mainly from 17.12 to 20.26. The content of MP in beer was not significantly different from that in the wort, primarily because MP does not participate in yeast metabolism [34]. The content of GP decreased from 0.41–0.46 mg/mL to 0.29–0.33 mg/mL. This decrease may be attributed to the fact that yeast can utilize GP for cellular metabolism during the brewing process [35]. In summary, the addition of wheat bran from 0% to 20% increased the content of WEAX in beer by 0.89 mg/mL, representing an increase of 65.44%. It also led to an increase in the content of WEAX, avDP, and A/X ratio in beer. However, there was no significant improvement in the content of AG, MP, and GP in beer.

#### 3.2.2. Changes in the Molecular Composition of Polysaccharides in Beer

**Figure 4:**
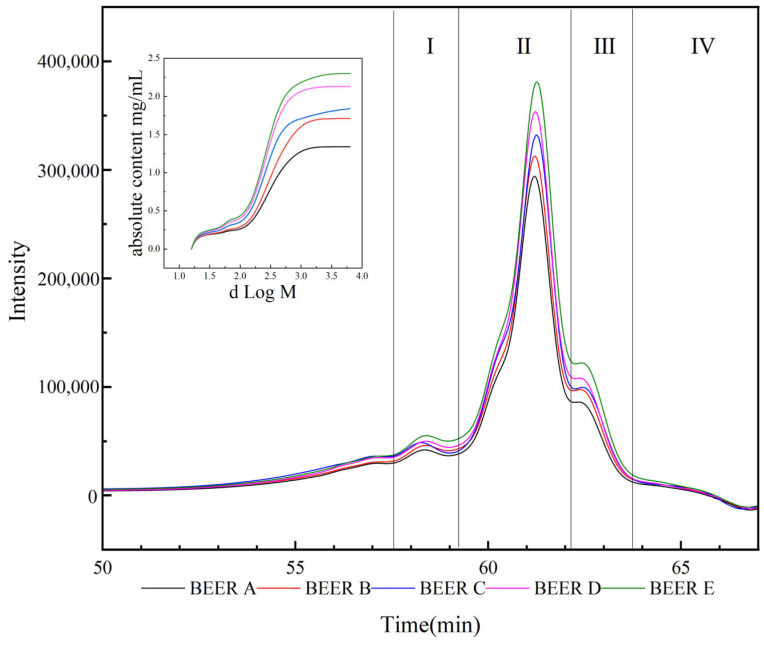
Molecular weight distribution and polysaccharide content in beer with different bran additions. I polysaccharide molecular weight 7062~10,134 Da; II polysaccharide molecular weight 2936~7062 Da; III polysaccharide molecular weight 859~2936 Da; IV polysaccharide molecular weight < 859 Da. BEER A: adding 0% wheat bran; BEER B: adding 5% wheat bran; BEER C: adding 10% wheat bran; BEER D: adding 15% wheat bran; BEER E: adding 20% wheat bran.

The impact of changes in wheat bran proportion on the molecular composition and content of polysaccharides in beer is depicted in Figure 4. Based on the peak shapes in the chromatogram, the polysaccharides in beer can be categorized into four components: I, II, III, and IV, with molecular weights ranging from 7062 to 10,134 Da, 2936 to 7062 Da, 859 to 2936 Da, and <859 Da, respectively. Component II is the predominant polysaccharide in beer, accounting for 63.10% to 66.02% of the polysaccharide content, with absolute content ranging from 0.86 to 1.49 mg/mL. The next significant components are III, I, and IV, which account for 15.88–16.76%, 11.27–13.29%, and 6.83–8.88% of the polysaccharide content, respectively. Their absolute content ranges from 0.15 mg/mL to 0.30 mg/mL, 0.23 to 0.36 mg/mL, and 0.12 to 0.16 mg/mL, respectively.

According to Table 5, the increase in peak heights of the four components in Figure 4 was mainly caused by the elevation of WEAX. By examining the specific content of WEAX in the five types of beer in Table 5 and the relative content of each component in Figure 4, it is possible to calculate the molecular composition and absolute increase in WEAX in beer, which was the result of the addition of wheat bran. The addition of wheat bran can lead to an increase in the content of WEAX in beer for the four components with molecular weights of 7062–10,134 Da, 2936–7062 Da, 859–2936 Da, and <859 Da, by approximately 0.06–0.15 mg/mL, 0.24–0.62 mg/mL, 0.05–0.13 mg/mL, and 0.01–0.04 mg/mL, respectively. Among them, the component with a molecular weight of 3119–7062 Da exhibited the largest increase in content.

By comparing Figure 2 to the molecular weight and content of polysaccharides in the wort, several observations can be made that the components with molecular weights of 8411–10,959 Da and 7062–8411 Da showed a significant decrease in content, ranging from 25.04% to 34.78%. This reduction may be attributed to two factors. First, larger polysaccharides may undergo precipitation and sedimentation during fermentation, leading to their decrease in the beer [36]. Secondly, yeast can utilize AG and preferentially consume AG side chains [35], resulting in a decrease in the content of this polysaccharide fraction. The content of components with molecular weights < 920 Da showed a decrease, ranging from 16.67% to 20.01%.

#### 3.2.3. Effect of Bran Addition on Physicochemical Indexes of Beer

The basic parameters of the beer are shown in Table 6; it can be found that the apparent attenuation of the beer slightly decreased with the increased wheat bran content. This is primarily due to the increased content of non-fermentable sugars present in the wort, specifically WEAX. The turbidity of the beer showed an increasing trend, with an improvement rate of up to 21.16%. As the wheat bran addition increased, the viscosity of the beer increased by 6.29% to 15.43%. Compared to the wort in Table 4, the beer exhibited a decrease in viscosity, and the reduction rate of viscosity increased with the enhancement in wheat bran addition, ranging from 10.71% to 14.77%. This may be attributed to the precipitation and sedimentation of polysaccharides with molecular weights of 8411–10,959 Da and 7062–8411 Da during fermentation [36], leading to a decrease in the viscosity of the beer. After fermentation, the pH of the beer decreased by 20.11% to 23.01%, while the total acidity increased by 4.46% to 18.25%. This is mainly attributed to the generation of organic acids like lactic acid, acetic acid, and malic acid through yeast fermentation metabolism. The generation of these organic acids led to a decreased pH of the wort [37].

#### 3.2.4. Effect of Bran Addition on the Rheological Properties of Beer

**Figure 5:**
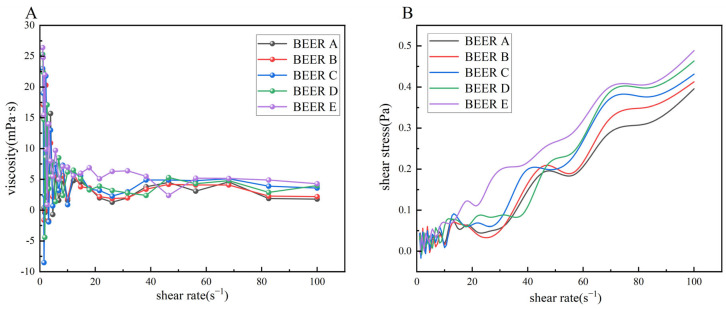
Rheological characterization of wheat-bran-added beers. (**A**) Shear-thinning behavior: apparent viscosity (mPa·s) vs. shear stress (s^−1^); (**B**) flow curve analysis: shear stress (Pa) vs. shear rate (s⁻^1^). Samples: BEER A (0% bran), B (5%), C (10%), D (15%), E (20%).

Figure 5 shows the beer’s shear viscosity and shear force with varying shear rates for different bran additions. It can be observed that, under identical shear rates, beer viscosity typically rises with increased bran content. This increase in viscosity is likely due to the higher WEAX content in the solution, which enhances interactions and entanglement of the polysaccharide chains, manifesting macroscopically as an increase in viscosity [38]. The viscosity of the beer decreased with increasing shear rate and remained almost constant at shear rates above 40 s⁻^1^. Meanwhile, as the shear rate increased, so did the shear stress. This indicated that even after the addition of bran, the beer exhibited shear thinning; its viscosity decreased when subjected to shear. This suggests that beer is a pseudoplastic fluid and a typical non-Newtonian fluid [39].

#### 3.2.5. Effect of Bran Addition on Foam Characteristics and Sensory Quality of Beer

**Figure 6:**
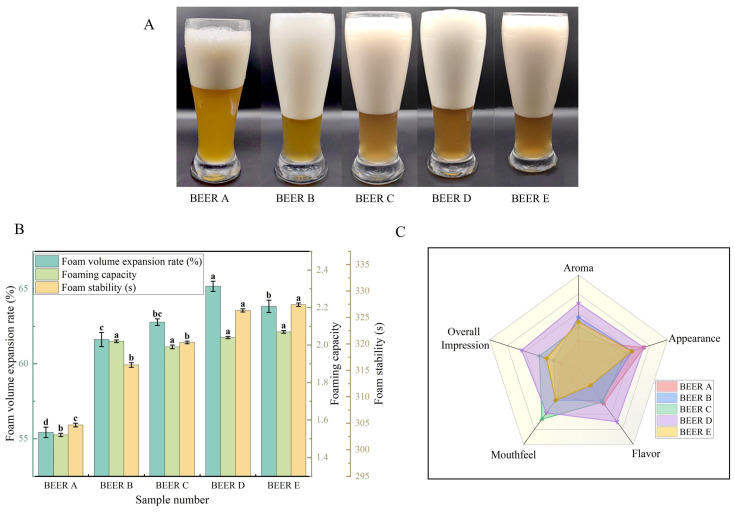
Foam properties and sensory characteristics of beer with different bran additions. (**A**) Appearance of beer foam; (**B**) foam holding time, foam retention, and foam expansion rate; (**C**) sensory radar charts. Samples: BEER A (0% bran), B (5%), C (10%), D (15%), E (20%). Difference letters indicate significant differences between beer samples with different bran additions (*p* < 0.05).

The foam characteristics and sensory quality of beer are depicted in Figure 6. Figure 6B illustrates that adding wheat bran, compared to beer without wheat bran, resulted in an increase from 6.02% to 7.47% in foam stability. Foam capacity was enhanced by 32.89% to 36.18%, and the foam volume expansion rate increased by 14.79% to 15.54%. These results suggest a notable enhancement in beer foam characteristics attributed to higher WEAX content and avDP. This enhancement can be attributed to the increased viscosity of the beer caused by WEAX, which slows down the rate of drainage from the foam and thus increases the foam’s stability [40]. However, within the range of wheat bran additions from 5% to 20%, there were no significant differences in foam capacity.

Moreover, incorporating wheat bran led to a rise in beer chroma by 16.52% to 50.82%. This indicates that the beer had a higher content of melanoidin, which is responsible for the dark chroma in beer. Wheat bran has been found to contain higher levels of phenolic substances [41], which contribute to the formation of melanoidins. It is known that melanoidins and polyphenols have a positive impact on foam stability in beer [42].

The sensory evaluation of beer is illustrated in Figure 6C. Combined with the appearance of the beer in Figure 6A, observations reveal that the chroma of the beer ranged from golden yellow to amber. BEER D shows the most pronounced aromatic profile. Except for BEER E, the beer had a pleasantly balanced and harmonious taste with a lingering aftertaste. BEER C had a moderate alcohol intensity and a moderate level of astringency. Previous studies have also shown that WEAX can affect the mouthfeel and viscosity of beer [43]. In terms of overall evaluation, BEER D was rated as the most enjoyable due to its outstanding appearance, aroma, and taste.

**Figure 7:**
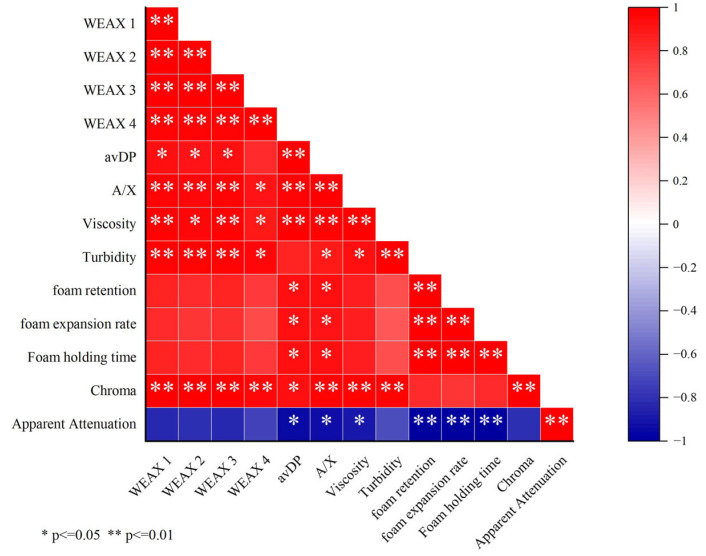
Heatmap of correlation among WEAX and physicochemical indicators of beer. Data analyzed by Pearson correlation (*p* < 0.05). Color scale indicates correlation strength from −1 (blue) to +1 (red). WEAX 1: molecular weight 7062~10,134 Da; WEAX 2: molecular weight 2936~7062 Da; WEAX 3: molecular weight 859~2936 Da; WEAX 4: molecular weight < 859 Da. A/X: arabinose/xylose; avDP: average degree of polymerization. Significance levels: * *p* < 0.05, ** *p* < 0.01.

The correlation between WEAX and the physicochemical parameters of beer is depicted in Figure 7; it can be observed that the viscosity of the beer showed a highly significant positive correlation with WEAX molecules with molecular weights ranging from 7062 to 10,134 Da and 859 to 2936 Da, with correlation coefficients of 0.963 and 0.962, respectively. Additionally, a notable positive correlation was observed among viscosity and WEAX molecules with molecular weights ranging from 2936 to 7062 Da and >859 Da, with correlation coefficients of 0.955 and 0.889, respectively. The turbidity of the beer exhibited a highly significant positive correlation with WEAX molecules with molecular weights ranging from 7062 to 10,134 Da, 2936 to 7062 Da, and 859 to 2936 Da, with correlation coefficients of 0.963, 0.971, and 0.966, respectively. The foam stability, foam volume expansion rate, and foaming capacity of the beer were all significantly positively correlated with the avDP and the A/X, with correlation coefficients of 0.937, 0.931, 0.940, 0.912, 0.937, and 0.931, respectively. The chroma of the beer exhibited a highly significant positive correlation with WEAX molecules of all four molecular weight ranges. The apparent attenuation of the beer exhibited a marked negative correlation with avDP and A/X of WEAX, with correlation coefficients of −0.952 and −0.927, respectively. Taken together, the WEAX molecules have a highly significant positive correlation with beer viscosity, turbidity, foam quality, and chroma, while avDP and A/X negatively correlate with the apparent attenuation.

## 4. Conclusions

The addition of 0–20% bran to pure wheat malt beer significantly reduced filtration times, with a 40% decrease observed at the 20% addition level. Bran primarily influenced the WEAX content without significantly affecting other polysaccharides such as arabinogalactan, mannan polysaccharide, and glucan polysaccharide. Specifically, the WEAX content in the molecular weight band of 2936–7062 Da increased by approximately 65.44%. Furthermore, higher WEAX content was positively correlated with beer turbidity and viscosity. Additionally, the degree of polymerization and the arabinose-to-xylose ratio of WEAX were found to positively affect foam stability, foam volume expansion rate, and foaming capacity.

## Figures and Tables

**Table 1:** Basic raw material index.

Index	Water (%)	Protein (%)	WEAX (%)		WUAX (%)	Total Sugar (%)	Lipid (%)	Ash (%)
Wheat bran	12.88 ± 0.12	17.36 ± 0.3	0.94 ± 0.03		15.80 ± 0.26	56.13 ± 0.85	2.49 ± 0.03	5.48 ± 0.12
Index	Water (%)	Saccharification time (min)	Total acid (mL/100 g)	pH	Protein (%)	Kolbach index (%)	Leaching rate (%)	FAN (mg/100 g)
Wheat malt	5.50 ± 0.08	10 ± 0	0.82 ± 0.03	6.03 ± 0.12	19.33 ± 0.13	38.10 ± 0.42	77.82 ± 0.99	139.31 ± 1.39

WEAX: water-extractable arabinoxylan. WUAX: water-unextractable arabinoxylan; Kolbach index: protein degradation efficiency. FAN: free amino nitrogen.

**Table 2:** Calibration curves of monosaccharides analyzed by GC for quantitative determination.

Monosaccharide	Chromatographic Peak	Retention Period (min)	Standard Curve	Correlation Coefficient
Ara	1	6.697	Y = 102082x − 7029.2	R^2^ = 0.9978
Xyl	2	6.934	Y = 92983x − 6066.2	R^2^ = 0.9981
Man	3	11.474	Y = 81169x − 5216.4	R^2^ = 0.9981
Gal	4	12.598	Y = 87185x − 5475.3	R^2^ = 0.9982
Glu	5	13.956	Y = 72644x − 4440.4	R^2^ = 0.9980

Ara: arabinose, Xyl: xylose, Man: mannose, Gal: galactose, Glu: glucose.

**Table 3:** Effect of bran addition on the composition and content of polysaccharides in wort.

	WEAX (mg/mL)	avDP	A/X	AG (mg/mL)	MP (mg/mL)	GP (mg/mL)
WORT A	1.41 ± 0.01 ^e^	17.42 ± 0.05 ^e^	0.52 ± 0.01 ^c^	0.41 ± 0.01 ^b^	0.23 ± 0.02 ^b^	0.37 ± 0.01 ^a^
WORT B	1.69 ± 0.03 ^d^	19.21 ± 0.03 ^d^	0.61 ± 0.01 ^b^	0.43 ± 0.01 ^ab^	0.25 ± 0.01 ^ab^	0.36 ± 0.00 ^a^
WORT C	1.94 ± 0.02 ^c^	20.05 ± 0.07 ^c^	0.62 ± 0.02 ^b^	0.43 ± 0.00 ^ab^	0.27 ± 0.00 ^a^	0.34 ± 0.00 ^ab^
WORT D	2.13 ± 0.01 ^b^	20.42 ± 0.02 ^b^	0.64 ± 0.01 ^ab^	0.44 ± 0.01 ^ab^	0.28 ± 0.00 ^a^	0.33 ± 0.02 ^b^
WORT E	2.31 ± 0.03 ^a^	20.76 ± 0.04 ^a^	0.66 ± 0.02 ^a^	0.46 ± 0.01 ^a^	0.29 ± 0.01 ^a^	0.33 ± 0.01 ^b^

Different lowercase letters indicate significant differences between groups (each column represents an experimental group) (*p* < 0.05). Samples: WORT A (0% bran), B (5%), C (10%), D (15%), E (20%). WEAX: water-extractable arabinoxylan, avDP: average degree of polymerization, A/X: arabinose/xylose ratio, AG: arabinogalactan, MP: mannan polysaccharide, GP: glucan polysaccharide. Different letters in the same column indicate significant differences between wort samples with different bran additions (*p* < 0.05).

**Table 4:** Effect of bran addition on physicochemical indexes of wort.

	WORT A	WORT B	WORT C	WORT D	WORT E
pH	5.42 ± 0.03 ^c^	5.45 ± 0.05 ^c^	5.53 ± 0.03 ^b^	5.55 ± 0.03 ^b^	5.65 ± 0.01 ^a^
Total acid (g/L)	1.47 ± 0.04 ^a^	1.42 ± 0.07 ^a^	1.29 ± 0.02 ^b^	1.27 ± 0.04 ^b^	1.12 ± 0.04 ^c^
Viscosity (mPa·s)	1.96 ± 0.04 ^c^	1.99 ± 0.02 ^c^	2.23 ± 0.04 ^b^	2.25 ± 0.02 ^b^	2.37 ± 0.05 ^a^
Chroma (EBC)	6.3 ± 0.1 ^e^	8.4 ± 0.1 ^d^	9.2 ± 0.0 ^c^	10.9 ± 0.0 ^b^	11.5 ± 0.1 ^a^
FAN (mg/100 g)	263.21 ± 7.50 ^a^	244.33 ± 8.02 ^b^	232.32 ± 7.29 ^b^	221.71 ± 3.43 ^c^	201.77 ± 8.21 ^d^
Protein (g/L)	18.64 ± 0.04 ^a^	17.00 ± 0.03 ^b^	15.03 ± 0.03 ^c^	14.27 ± 0.01 ^d^	13.52 ± 0.02 ^e^
Saccharification time (min)	8 ± 0 ^b^	8 ± 0 ^b^	10 ± 0 ^a^	10 ± 0 ^a^	10 ± 0 ^a^
Filtration time (min)	40 ± 3 ^a^	32 ± 2 ^b^	28 ± 2 ^b^	25 ± 1 ^c^	22 ± 1 ^d^
Original Gravity (°P)	12.33 ± 0.93 ^a^	12.32 ± 0.81 ^a^	12.34 ± 0.77 ^a^	12.30 ± 0.21 ^a^	12.34 ± 0.63 ^a^

The means within a line with different small letters (a–e) are significantly different (*p* < 0.05). Samples: WORT A (0% bran), B (5%), C (10%), D (15%), E (20%). FAN: free amino nitrogen. Different letters in the same row indicate significant differences between wort samples with different bran additions (*p* < 0.05).

**Table 5:** Effect of bran addition on the composition and content of polysaccharides in beer.

	WEAX (mg/mL)	avDP	A/X	AG (mg/mL)	MP (mg/mL)	GP (mg/mL)
BEER A	1.36 ± 0.02 ^e^	17.12 ± 0.01 ^e^	0.53 ± 0.00 ^d^	0.29 ± 0.01 ^b^	0.24 ± 0.01 ^a^	0.33 ± 0.03 ^a^
BEER B	1.67 ± 0.03 ^d^	18.96 ± 0.04 ^d^	0.60 ± 0.00 ^c^	0.31 ± 0.02 ^ab^	0.25 ± 0.00 ^a^	0.33 ± 0.04 ^a^
BEER C	1.89 ± 0.02 ^c^	19.92 ± 0.03 ^c^	0.62 ± 0.01 ^b^	0.31 ± 0.01 ^ab^	0.24 ± 0.01 ^a^	0.31 ± 0.03 ^a^
BEER D	2.10 ± 0.02 ^b^	20.02 ± 0.06 ^b^	0.63 ± 0.00 ^b^	0.32 ± 0.00 ^a^	0.25 ± 0.00 ^a^	0.30 ± 0.02 ^a^
BEER E	2.25 ± 0.01 ^a^	20.26 ± 0.04 ^a^	0.66 ± 0.01 ^a^	0.33 ± 0.01 ^a^	0.26 ± 0.01 ^a^	0.30 ± 0.02 ^a^

The means within a column with different small letters (a–e) are significantly different (*p* < 0.05). Samples: BEER A (0% bran), B (5%), C (10%), D (15%), E (20%) WEAX: water-extractable arabinoxylan, avDP: average degree of polymerization, A/X: arabinose/xylose ratio, AG: arabinogalactan, MP: mannan polysaccharide, GP: glucan polysaccharide. Different letters in the same column indicate significant differences between beer samples with different bran additions (*p* < 0.05).

**Table 6:** Effect of bran addition on physicochemical indexes of beer.

	BEER A	BEER B	BEER C	BEER D	BEER E
Original Gravity (°P)	12.40 ± 1.01 ^a^	12.37 ± 0.93 ^a^	12.41 ± 0.77 ^a^	12.37 ± 0.80 ^a^	12.41 ± 0.92 ^a^
Apparent Attenuation (%)	69.57 ± 0.03 ^a^	67.60 ± 0.02 ^b^	67.47 ± 0.02 ^c^	67.43 ± 0.01 ^cd^	67.39 ± 0.01 ^d^
Alcohol (%)	5.31 ± 0.02 ^a^	5.27 ± 0.01 ^b^	5.26 ± 0.01 ^b^	5.24 ± 0.02 ^bc^	5.21 ± 0.03 ^c^
pH	4.33 ± 0.02 ^b^	4.32 ± 0.01 ^b^	4.38 ± 0.01 ^a^	4.34 ± 0.02 ^b^	4.35 ± 0.01 ^b^
Total acid (g/L)	1.54 ± 0.01 ^a^	1.43 ± 0.01 ^b^	1.40 ± 0.02 ^b^	1.39 ± 0.02 ^b^	1.37 ± 0.01 ^b^
Viscosity (mPa.s)	1.75 ± 0.02 ^c^	1.76 ± 0.01 ^c^	1.96 ± 0.00 ^b^	1.97 ± 0.01 ^b^	2.02 ± 0.00 ^a^
Chroma (EBC)	5.5 ± 0.2 ^e^	6.4 ± 0.1 ^d^	7.1 ± 0.3 ^c^	7.6 ± 0.2 ^b^	8.3 ± 0.1 ^a^
Turbidity (EBC)	4.49 ± 0.06 ^e^	4.61 ± 0.09 ^d^	4.92 ± 0.07 ^c^	5.18 ± 0.07 ^b^	5.44 ± 0.09 ^a^
FAN (mg/100 g)	81.68 ± 0.04 ^a^	74.48 ± 0.02 ^b^	67.37 ± 0.03 ^c^	63.47 ± 0.01 ^d^	54.89 ± 0.03 ^e^
Protein (g/L)	13.07 ± 0.00 ^a^	11.39 ± 0.01 ^b^	11.27 ± 0.02 ^c^	10.21 ± 0.01 ^d^	9.77 ± 0.01 ^e^

The means within a line with different small letters (a–e) are significantly different (*p* < 0.05). Samples: BEER A (0% bran), B (5%), C (10%), D (15%), E (20%). FAN: free amino nitrogen. Different letters in the same row indicate significant differences between beer samples with different bran additions (*p* < 0.05).

## Data Availability

The original contributions presented in the study are included in the article, further inquiries can be directed to the corresponding author.
